# Sulfonamide Inhibition Profile of the β-Carbonic Anhydrase from *Malassezia restricta*, An Opportunistic Pathogen Triggering Scalp Conditions

**DOI:** 10.3390/metabo10010039

**Published:** 2020-01-16

**Authors:** Sonia Del Prete, Andrea Angeli, Cynthia Ghobril, Julien Hitce, Cécile Clavaud, Xavier Marat, Claudiu T. Supuran, Clemente Capasso

**Affiliations:** 1 Institute of Biosciences and Bioresources, CNR, Via Pietro Castellino 111, 80131 Napoli, Italy; sonia.delprete@ibbr.cnr.it; 2 Section of Pharmaceutical and Nutraceutical Sciences, Department of Neurofarb, University of Florence, Via U. Schiff 6, 50019 Sesto Fiorentino, Florence, Italy; andrea.angeli@unifi.it; 3 L’Oréal Research and Innovation, 93601 Aulnay-sous-Bois, France; cynthia.ghobril@rd.loreal.com (C.G.); julien.hitce@rd.loreal.com (J.H.); cecile.clavaud@rd.loreal.com (C.C.); xavier.marat@rd.loreal.com (X.M.)

**Keywords:** carbonic anhydrases, metalloenzymes, sulfonamides, CA inhibitors, *Malassezia restricta*, *Malassezia globosa*, dandruff, seborrheic dermatitis

## Abstract

The critical CO_2_ hydration reaction to bicarbonate and protons is catalyzed by carbonic anhydrases (CAs, EC 4.2.1.1). Their physiological role is to assist the transport of the CO_2_ and HCO_3_^−^ at the cellular level, which will not be ensured by the low velocity of the uncatalyzed reaction. CA inhibition may impair the growth of microorganisms. In the yeasts, *Candida albicans* and *Malassezia globosa*, the activity of the unique β-CA identified in their genomes was demonstrated to be essential for growth of the pathogen. Here, we decided to investigate the sulfonamide inhibition profile of the homologous β-CA (MreCA) identified in the genome of *Malassezia restricta,* an opportunistic pathogen triggering dandruff and seborrheic dermatitis. Among 40 investigated derivatives, the best MreCA sulfonamide inhibitors were dorzolamide, brinzolamide, indisulam, valdecoxib, sulthiam, and acetazolamide (K_I_ < 1.0 μM). The MreCA inhibition profile was different from those of the homologous enzyme from *Malassezia globosa* (MgCA) and the human isoenzymes (hCA I and hCA II). These results might be useful to for designing CA inhibitor scaffolds that may selectively inhibit the dandruff-producing fungi.

## 1. Introduction

The scalp has a high follicular density and an enhanced sebum production rate compared to other human skin areas [1]. Dandruff begins with puberty with a peak incidence at 20 years of age and is less prevalent over 50 years [2], linked with sebaceous gland activity [3]. Dandruff has been considered as a less severe form of scalp seborrheic dermatitis, characterized by skin flacking, sub-inflammation, pruritus, and absence of erythema. Both scalp conditions are related to higher levels of the lipophilic yeasts belonging to the genera *Malassezia* (e.g., *M. globosa* and *M. restricta*) [1,2]. These fungi, through their secreted enzyme lipase, digest the sebaceous triglycerides of the skin, producing saturated fatty acids, essential for the growth of the parasites, as well as unsaturated acids (e.g., oleic acid and arachnoid acid), which provoke irritation and inflammation of the skin, respectively [4]. *Malassezia* species are also able to change the natural release of dead skin cells. Furthermore, a disequilibrium of the scalp bacteria population, such as *Propionibacterium acnes* and *Staphylococcus epidermidis,* as well as non-microbial factors, like the break of the scalp, may trigger dandruff [5,6]. Treatment of *Malassezia*-related dermatosis generally requires the use of topical or oral antifungal drugs, often in combination with antifungal washes and shampoos. In particular, the treatment of dandruff is done through the use of zinc pyrithione, selenium sulfide, ketoconazole, climbazole, clotrimazole, piroctone olamine, keratolytic agent (salicylic acid), anti-proliferative agent (Coal Tar) [2,7]. All of these traditional drugs, including shampoos, creams, and lotions, have been used for years showing effectiveness, which is however limited to the duration of the treatment, and relapse occurs 3 to 6 weeks after the treatment is stopped [2]. We recently proposed exploring certain members of the ancient superfamily of ubiquitous metalloenzymes, known as carbonic anhydrases (CAs, EC 4.2.1.1) from *Malassezia restricta*, as a new antidandruff target [8,9]. CAs catalyze the simple but physiologically crucial reaction of CO_2_ hydration to bicarbonate and protons (CO_2_ + H_2_O ⇄ HCO_3_^−^ + H^+^) [10] and they are the fastest among the known enzymes (k_cat_ = 10^4^–10^6^ s^−1^) [11]. CAs are currently categorized into eight genetically distinct families (or classes), named with the Greek letters: α-, β-, γ-, δ-, ζ-, η, θ, and ι [12]. The last three classes were only recently discovered [13,14]. All catalytically active CAs, independently of the genetic class, contain a metal ion in the active site, generally Zn(II), which is essential for catalysis [11,15,16,17]. The proposed physiological role of CAs is to assist the transport of carbon dioxide and bicarbonate, making possible their balance inside the cell and supplying demand of CO_2_ and bicarbonate to the central metabolism, which will not be ensured by the very low k_cat_ (0.15 s^−1^) of the uncatalyzed CO_2_ hydration/dehydration reaction [8,18,19,20,21,22]. It is readily understandable that CA inhibition may impair the growth of microorganisms, altering the CO_2_ and bicarbonate balance [22]. The β-CA identified in the genome of *Candida albicans* is responsible, together with adenylyl cyclase, for the conversion between the yeast and filamentous growth phases, in response to ambient CO_2_ level [23]. In 2012, a β-CA (acronym MgCA) from the fungal pathogen *Malassezia globosa*, one of the fungi responsible for dandruff, was efficiently inhibited in vitro by the classical CA inhibitors (CAIs), such as sulfonamides, sulfamates, and sulfamides with K_Is_ in the nanomolar to the micromolar range [24]. Furthermore, the susceptibility tests performed on different species of *Malassezia* (*M. dermatis*, *M. furfur*, *M. pachydermatis*, and *M. globosa*) demonstrated that several sulfonamides were able to inhibit their growth [24]. Finally, a dandruff mouse model revealed that treatment with sulfonamides resulted in fragmented fungal hyphae, as occurs with ketoconazole, a clinically-used antifungal agent [24]. In this context, we decided to investigate the sulfonamide inhibition profile of the homologous β-CA (acronym MreCA) recently identified in the genome of *M. restricta* [8,18], which cooperates with *M. globosa* and the bacterial scalp in triggering dandruff and seborrheic dermatitis [25,26]. The MreCA sulfonamide inhibition profile was compared with those reported for the two human α-CA isoforms (hCA I and hCA II) and the β-CA from *M. globosa* with the intent to select new potential anti-dandruff and anti-seborrheic dermatitis compounds.

## 2. Results and Discussion

The exploration of the fungal genomes evidenced the presence of a variegated distribution pattern of the CA-classes. The genome of filamentous ascomycetes encodes for 𝛼- and β-classes, while saccharomycetes and basidiomycetes genomes contain only β-CAs [27,28]. Furthermore, the fungal genomes may have multiple forms of the same gene. For example, the ascomycetes’ genome contains three isoforms of β-CAs and at least one 𝛼-CA; the genome of basidiomycetes, such as *Cryptococcus neoformans,* have two β-CAs, while the genomes of *Coprinopsis cinereal* and *Ustilago maydis* contain a single β-CA gene. All fungal CAs, alone or in association with the soluble adenylate cyclase, are involved in the cellular balance of CO_2_ and bicarbonate, CO_2_-sensing, and regulation of fungal sexual development [29,30,31,32]. The inhibition of the fungal CAs may alter the metabolism of the fungus, impairing its growth and virulence [22]. A variety of CA inhibitors (CAIs) are known, such as the metal complexing anions, and the unsubstituted sulfonamides [33]. They bind to the Zn(II) ion of the enzyme either by substituting the non-protein zinc ligand or by adding to the metal coordination sphere generating trigonal-bipyramidal species [33]. CAIs belonging to sulfonamide, thiol or DTC were able to inhibit the growth of *M. globosa*, *C. albicans*, *Cryptococcus neoformans in vivo,* and in the case of the dandruff associated fungus, *M. globosa*, led to very effective control of the infection in an animal model [19,20,21,22,34,35,36]. In addition, as β-CAs are not present in human cells, the adverse effects arising from host enzyme inhibition might be limited. In this context, an extensive sulfonamide *in vitro* inhibition study was carried out on the recombinant β-CA (MreCA) encoded by the genome of the fungus *M. restricta*, which represents the larger proportion of *Malassezia sp.* on the human scalp.

### 2.1. Integrity of the Target Enzyme

**Figure 1:**
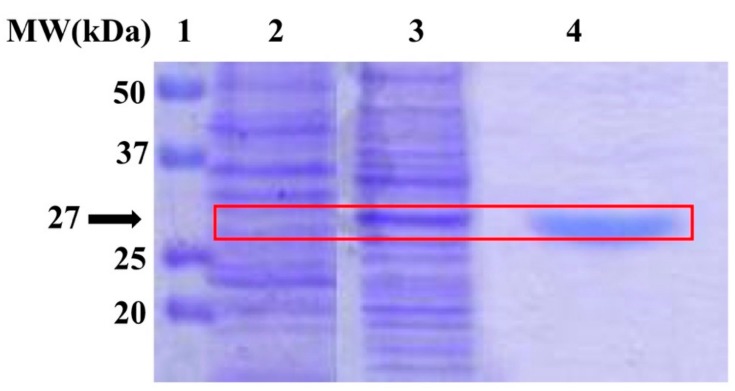
SDS-PAGE analysis. The overexpression of the MreCA fusion protein was obtained using 1 mM IPTG. The overexpressed fungal CA is well evident in lane 2 at the molecular weight of 27 kDa, while it was absent in lane 1, which contained the cell lysate supernatant before IPTG induction. After induction, the soluble MreCA was passed through the HisTrap FF column to obtain a highly pure and homogenous MreCA. Legend: Lane 1, molecular markers; Lane 2, cell lysate supernatant; Lane 3, IPTG induction; Lane 4, purified MreCA. The red box identifies the height in the gel corresponding to 27 kDa.

The recombinant MreCA was heterologously produced using *E. coli* as a host [8]. The integrity of the overexpressed recombinant MreCA was verified through the use of two biochemical techniques, SDS-PAGE and protonography. The latter is specific for the detection of the CO_2_ hydratase activity on the polyacrylamide gel. Figure 1 shows the SDS–PAGE carried out on the supernatant of the cell lysate before and after the induction of isopropyl β-D-1-thiogalactopyranoside (IPTG), as well as the pure enzyme obtained by the affinity column. The SDS-PAGE analysis revealed that the cells induced with 1 mM IPTG overexpressed the MreCA fusion protein at the expected size (27 kDa) (Figure 1). This result confirms that MreCA was heterologously produced in the cytoplasm as a soluble protein after IPTG induction. The MreCA fusion protein containing the *N*-terminal (His)_6_-tag was purified to homogeneity, loading the supernatant of the cell lysate on an affinity HisTrap FF column (Figure 1).

**Figure 2:**
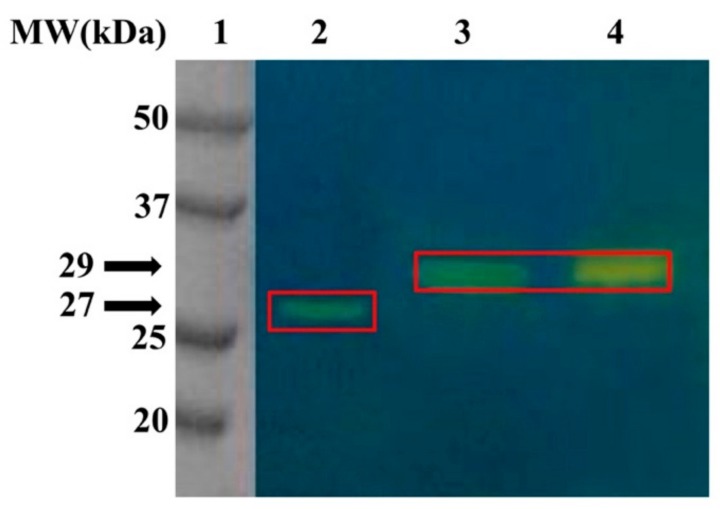
Protonography analysis. Developed protonogram showing the CO_2_ hydratase activity of MreCA, MgCA, and bCA directly on the SDS-PAGE. Legend: Lane 1, molecular markers; Lane 2, purified MreCA; Lane 3 and 4, purified MgCA and commercial bovine CA, respectively. These two enzymes were used as positive controls. The red boxes identified the yellow bands at 27 and 29 kDa.

To verify whether the purified recombinant MreCA was also able to perform the CO_2_ hydration reaction, the fungal CA was subjected to the protonography analysis (Figure 2). To accomplish this technique, after the run, the SDS-PAGE gel was treated with blue bromothymol, which appears blue in its deprotonated form. The production of H^+^ ions, due to the CA hydratase activity, lowers the pH of the solution to pH 6.8, the color transition point of the dye, developing a yellow band at a molecular weight of the CA. As a comparison, β-CA (MgCA) from *M. globosa* and the commercial bovine 𝛼-CA (bCA) have been used. As expected, the protonogram (Figure 2) shows the yellow colors, corresponding to the CO_2_ hydratase activity, at the gel position corresponding to 27 kDa, the molecular weight of the recombinant MreCA, and 29 kDa (MgCA and bCA molecular weight). The development of the protonogram requires the elimination of the SDS from the gel to detect the enzyme activity. The three enzymes, MreCA, MgCA, and bCA, were able to refold and generate their active form correctly. This is typical of other CA classes present in prokaryotic/eukaryotic organisms [37].

The CO_2_ hydratase activity of the purified and soluble enzyme, as well as the kinetic constants, were determined using the stopped-flow technique. The results were compared with those obtained for other fungal CAs (Table 1). The enzyme had high catalytic activity for the physiological reaction of CO_2_ hydration to bicarbonate and protons, with a k_cat_ of 1.06 × 10^6^ s^−1^ and a catalytic efficiency (k_cat_/K_M_) of 1.07 × 10^8^ M^−1^·s^−1^. The k_cat_ value of the recombinant MreCA resulted in one order higher than those calculated for the other fungal CAs [24,32,34,38], as well as for the human isoform, hCA I (𝛼-CA) [39,40]. Moreover, it was more active than the homologous MgCA enzyme. Intriguing, MreCA kinetic values are very similar to that of the human 𝛼-CA, the isoform hCA II, which is considered among the fastest CAs known.

### 2.2. Sulfonamide Inhibition Profile

**Figure 3:**
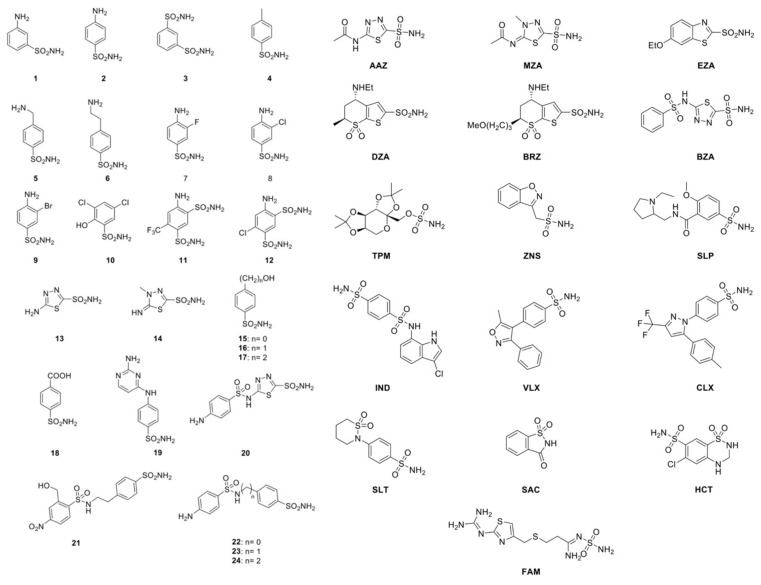
The 40 compounds. Thirty-nine sulfonamides and one sulfamate (TPM) were used to determine the MreCA inhibition profile.

With regards to their binding mode to the enzyme active site, CAIs may be classified into several different groups [33,41]. They include: (1) metal ion binders (anions, sulfonamides and their bioisosteres; dithiocarbamates, xanthates, etc.); (2) compounds that anchor to the water molecule/hydroxide ion coordinated to zinc (phenols, polyamines, thioxocoumarins, sulfocumarins); (3) compounds such as coumarins and their isosteres, which occlude the active site entrance; (4) compounds binding out of the active site, such as an aromatic carboxylic acid derivative [41]; and (5) inhibitors with an unknown binding mechanism, such as secondary/tertiary sulfonamides, protein tyrosine kinase inhibitors, and fullerenes, for which the X-ray crystallographic structure is unavailable [41]. Recently, it has been demonstrated that MreCA was efficiently inhibited by a large number of inorganic metal-complexing anions, such as diethyldithiocarbamate, sulfamide, phenyl arsenic acid, stannate, tellurate, tetraborate, selenocyanate, trithiocarbonate, and bicarbonate. Although most of these small inorganic drugs are not used in medicine, they may help to design novel types of inhibitors, which may have clinical applications. In this context, we investigated a rather large number of sulfonamides and their bioisosteres for their interaction with MreCA. A library of 40 compounds, 39 primary sulfonamides and one sulfamate, were used as CAIs [42,43,44,45,46,47,48,49,50,51,52,53,54,55,56,57]. Figure 3 indicates the molecular structures of these compounds.

Derivatives **1–24** and **AAZ-HCT** are either simple aromatic or heterocyclic sulfonamides that are widely used as building blocks for obtaining new potent and selective families of such pharmacological agents. The acronyms and the commercial names of the group **AAZ-HTC** are reported in Table 2.

**AAZ**, **MZA**, **EZA**, and **DCP** are the classical, systemically acting antiglaucoma CAIs. **DZA** and **BRZ** are topically acting antiglaucoma agents; **BZA** is an orphan drug belonging to this class of pharmacological agents. **ZNS**, **SLT**, and the sulfamic acid ester **TPM** are widely used antiepileptic drugs. **SLP** and **IND** were also shown by our group to belong to this class of pharmacological agents, together with the COX2 selective inhibitors **CLX** and **VLX**. **SAC** and the diuretic **HCT** are also known to act as CAIs. Sulfonamides, such as the clinically used derivatives **AAZ**, **MZA**, **EZA**, **DCP**, **DZA**, and **BZA**, bind in a tetrahedral geometry to the Zn(II) ion in the deprotonated state, with the nitrogen atom of the sulfonamide moiety coordinated to Zn(II) and an extended network of hydrogen bonds, involving amino acid residues of the enzyme, also participating in the anchoring of the inhibitor molecule to the metal ion [30,33,41,58]. The aromatic/heterocyclic part of the inhibitor interacts with the hydrophilic and hydrophobic residues of the catalytic cavity [41,58,59,60].

Table 3 includes the inhibition data of the two fungal enzymes, MreCA and MgCA [24]. Mainly, for comparison reasons, the data of the sulfonamide inhibition profiles of the two human isoforms, hCA I and hCA II, have also been added [39,40].

The following results can be observed from the data of Table 3:

1. Many of the investigated compounds, such as **3**, **5**, **6**, **8**, **9**, **10**, **12**, **13**, **14**, **15**, **17**, **18**, **23**, **24**, **ZNS**, and **FAM**, showed a weak MreCA inhibitory activity, with an inhibition constant (K_I_) higher than 10 μM. This is remarkable because most of these inhibitors block the human isoenzyme (hCA II) and the homologous fungal enzyme MgCA rather effectively. For example, **3**, **5**, **6**, **8**, **9**, **10**, **12**, **15, 17,** and **18** showed a K_I_ in the nanomolar range of 63–174 nM for MgCA, while hCA II, with these compounds, was inhibited with a K_I_ in the range of 8–170 nM. Intriguingly, hCA I was effectively inhibited by only two compounds on this list (**3** and **18**), with a K_I_ in the range 68–79 nM.
2. Several compounds of the series **1–24**, such as **1**, **2**, **4**, **7**, **11**, **16**, **19**, **21**, and **22**, had a moderate inhibitory effect on the MreCA, showing a K_I_ between 3.74–7.79 μM. Most of these inhibitors were potent inhibitors of hCA II (K_I_ = 11–300 nM) and weak inhibitors of hCA I (K_I_ = 5.8–28 μM), except for compounds 19, 21, and 22 (K_I_ = 16.4–109 nM). Similar behavior was shown by numerous clinically used compounds belonging to the series **AZZ-HTC**, such as **MZA**, **EZA**, **BZA**, **TPM**, **SLP**, **CLX**, **SAC**, **HTC**, and **DCP**. For these inhibitors, an inhibition constant ranging from 3.06 to 8.5 μM has been determined. Intriguingly, most of them are strong inhibitors of the two human isoenzymes (hCA I and hCA II), while most of those inhibitors, which resulted in moderate inhibitors of MreCA, were mild inhibitors of MgCA and vice-versa.
3. Among all the compounds investigated for MreCA inhibition, only seven of them showed inhibition constants of < 1.0 μM. This is the case for inhibitors **20**, **DZA**, **BRZ**, **IND**, **VLX**, **SLT,** and **AAZ**. These compounds had a K_I_ in the range of 0.1–0.91 μM. It is interesting to note that the MreCA “strong inhibitors” were mild inhibitors of MgCA (K_I_ = 31.5–79 μM), except for compound **20** (Table 3).
4. As shown in Table 3, the two homologous fungal enzymes have an inhibition pattern very different from each other. Furthermore, the inhibition profiles of MreCA and MgCA were highly distinct from those of the two human isoenzymes.

## 3. Materials and Methods

### 3.1. Chemicals and Instruments

IPTG and antibiotics were purchased from Sigma. The affinity column (His-Trap FF) and molecular weight markers were from GE Healthcare. All other chemicals used in this study were of reagent grade. The AKTA-prime purification system was purchased by GE Healthcare. The SX20 Stopped-Flow was obtained from Applied Photophysics, while the SDS–PAGE apparatus was procured by BioRAD.

### 3.2. Enzyme Integrity Determination

The synthetic *M. restricta* gene cloned into the expression vector pET100D-Topo/MreCA was used to transform the competent *Escherichia coli* BL21 (DE3) codon plus cells (Agilent) as reported by Del Prete et al. [8]. The cellular culture was induced with isopropyl β-D-1-thiogalactopyranoside (IPTG) to overexpress the recombinant MreCA. After the growth, the cells were harvested and disrupted by sonication. Cellular extract was purified using a nickel affinity column (His-Trap FF). The HisTrap column (1 mL) was equilibrated with 20 mL equilibration buffer (50 mM Tris, 20 mM imidazole and 150 mM sodium chloride, pH 7.5) at 1 mL/min. The supernatant from the cellular lysate was loaded onto the column at 1 mL/min, and connected with AKTA Prime. The recombinant MreCA was eluted from the column with a flow of 0.5 mL/min and the elution buffer was composed of 50 mM Tris, 500 mM imidazole and 300 mM sodium chloride, at pH 7.5. The recovered MreCA was 90% pure. The protein quantification was carried out by Bradford method (BioRAD) [61]. The enzyme integrity was verified using SDS-PAGE and the protonography. A 12% sodium dodecyl sulfate-polyacrylamide gel electrophoresis (SDS-PAGE) was prepared as described by Laemmli [62], and run by loading the supernatant from the cellular extract before and after induction with IPTG, and the recovered MreCA from the affinity column, on the gel. The gel was stained with Coomassie Brilliant Blue-R. To perform the protonography, wells of 12% SDS-PAGE gel were loaded with MreCA, MgCA and bCA mixed with loading buffer without 2-mercaptoethanol and without boiling the samples, in order to avoid protein denaturation. The gel was run at 150 V until the dye front ran off the gel. Following the electrophoresis, the 12% SDS-PAGE gel was subject to protonography to detect the yellows bands due to the hydratase activity on the gel as described by Capasso and coworkers [37,63,64,65].

### 3.3. Determination of the Kinetic Parameters and Inhibition Constants

The CO_2_ hydration activity performed by the MreCA was monitored using an Applied Photophysics stopped-flow instrument [66]. Phenol red (at a concentration of 0.2 mM) was used as an indicator, working at the absorbance maximum of 557 nm, with 20 mM TRIS (pH 7.5) as buffer, and 20 mM NaClO_4_ (for maintaining the ionic strength at a constant level), following the initial rates of the CA-catalyzed CO_2_ hydration reaction for a period of 10–100 s. To determine the kinetic parameters by Lineweaver–Burk plots and the inhibition constants, a concentration of CO_2_ between 1.7 to 17 mM was used. At least six measurements of the original 5–10% reaction were used to assess the initial velocity for each inhibitor. The uncatalyzed rates were identically determined and detracted from the total observed rates. Stock inhibitor solutions (10–100 mM) were prepared in distilled-deionized water and dilutions up to 0.01 mM were done with the buffer test. Inhibitor and enzyme solutions were preincubated together for 15 min at room temperature prior to assay, in order to allow for the formation of the E-I complex or for the eventual active site mediated hydrolysis of the inhibitor. The inhibition constants, which represent the mean from at least three different determinations, were obtained by the non-linear least-squares methods using PRISM 6 and the Cheng–Prusoff equation, as reported earlier [67,68,69]. All CA isoforms were recombinant ones obtained in-house. All salts/small molecules were of the highest purity available, from Sigma-Aldrich (Milan, Italy).

## 4. Conclusions

The recombinant fungal MreCA, heterologously overexpressed using *E. coli* cells as hosts, was produced as a soluble cytoplasmic protein. Its integrity was checked using SDS-PAGE and protonography. The protein showed significant catalytic activity for the CO_2_ hydration reaction. The MreCA sulfonamide inhibition profile obtained using **1–24** simple aromatic/heterocyclic compounds and the clinically used drugs **AAZ-HTC**, showed these compounds to behave as weak or moderate inhibitors compared to the enzyme. Among all the investigated compounds, only seven of them (**20**, **DZA**, **BRZ**, **IND**, **VLX**, **SLT,** and **AAZ)** showed an inhibition constant (K_I_) < 1.0 μM. The most exciting aspect of this study comes from the comparison of the inhibition profiles of the two fungal enzymes and the two human isoenzymes (hCA I and hCA II). From the presented results, it is readily apparent that MreCA not only has an inhibition pattern utterly different from that of its homologous MgCA, but it is very different from those of the human CAs, belonging to a different class (𝛼-class). These results can be explained because the fungal (MreCA and MgCA) and human CAs (hCA I and hCAII) have a similar catalytic site, but unusual architectural features. At the moment, the crystallographic structures of the two fungal enzymes are not available. It is possible to speculate that the structural characteristics of each biocatalyst occurring in the interaction between the protein and thirty-nine sulfonamides and one sulfamate (TPM) investigated are responsible for the differences in K_I_ values obtained for the four enzymes studied. Furthermore, these data prompt us to modify the scaffold of the investigated inhibitors for finding new and selective antidandruff drug targets.

## Figures and Tables

**Table 1:** MreCA kinetic parameters compared with those calculated for the two human isoforms hCA I and II (α-class), and the fungal β-CAs from different species. The CO_2_ hydration reaction was followed at 25 °C, in 20 mM Tris buffer and 20 mM NaClO_4_, pH 8.3.

Organisms	Acronym	Class	K_cat_ (s^−1^)	k_cat_/K_M_ (M^−1^·s^−1^)
*Homo sapiens*	hCA I ^a^	𝛼	2.0 × 10^5^	5.0 × 10^7^
	hCA II ^a^	𝛼	1.4 × 10^6^	1.5 × 10^8^
*Malassezia retricta*	MreCA	β	1.06 × 10^6^	1.07 × 10^8^
*Malassezia globosa*	MgCA ^b^	β	9.2 × 10^5^	8.3 × 10^7^
*Cryptococcus neoformans*	Can2 ^c^	β	3.9 × 10^5^	4.3 × 10^7^
*Candida albicans*	CaNce103 ^d^	β	8.0 × 10^5^	9.7 × 10^7^
*Candida glabrata*	CgNce103 ^e^	β	3.8 × 10^5^	4.8 × 10^7^

^a^From ref. 39; ^b^ From ref. 24; ^c^ From ref. 32; ^d^ From ref. 34; ^e^ From ref. 38.

**Table 2:** Acronyms and commercial names of the CAI clinically used drugs.

CAI Acronym	Commercial Name
**AAZ**	Acetazolamide
**MZA**	Methazolamide
**EZA**	Ethoxzolamide
**DCP**	Dichlorophenamide
**DZA**	Dorzolamide
**BRZ**	Brinzolamide
**BZA**	Benzolamide
**ZNS**	Zonisamide
**SLT**	Sulthiame
**FAM**	Famotidine
**TPM**	Topiramate
**SLP**	Sulpiride
**IND**	Indisulam
**CLX**	Celecoxib
**VLX**	Valdecoxib
**SAC**	Saccharin
**HCT**	Hydrochlorothiazide

**Table 3:** Inhibition data of human isoenzymes (CA I and CA II) and fungal CAs (MreCA and MgCA) with the thirty-nine sulfonamides and one sulfamate by a stopped-flow CO_2_ hydrase assay.

K_I_ (µM) *				
Compound	MreCA	MgCA ^a^	hCA I ^b^	hCA II ^b^
**1**	4.12	9.8	28.0	0.300
**2**	4.62	0.245	25.0	0.240
**3**	>10	0.152	0.079	0.008
**4**	4.04	6.74	78.5	0.320
**5**	>10	0.174	25.0	0.170
**6**	>10	0.079	21.0	0.160
**7**	4.59	0.116	8.3	0.060
**8**	>10	0.121	9.8	0.110
**9**	>10	0.349	6.5	0.040
**10**	>10	0.543	7.3	0.054
**11**	6.76	0.09	5.8	0.063
**12**	>10	0.092	8.4	0.075
**13**	>10	79.0	8.6	0.060
**14**	>10	85.0	9.3	0.019
**15**	>10	0.236	5.5	0.080
**16**	6.51	0.104	9.5	0.094
**17**	>10	0.063	21.0	0.125
**18**	>10	0.068	0.164	0.046
**19**	7.79	35.0	0.109	0.033
**20**	0.91	0.234	0.006	0.002
**21**	7.40	0.118	0.069	0.011
**22**	3.74	0.094	0.0164	0.046
**23**	>10	45.3	0.109	0.033
**24**	>10	2.56	0.095	0.030
**MZA**	3.90	74.55	0.05	0.014
**EZA**	3.79	38.0	0.025	0.008
**DZA**	0.81	79.0	50.0	0.009
**BRZ**	0.70	84.0	45.0	0.003
**BZA**	7.15	0.482	0.015	0.009
**TPM**	3.83	1.46	0.25	0.01
**SLP**	4.85	0.32	1.2	0.04
**IND**	0.87	n.d.	0.031	0.015
**ZNS**	>10	7.65	0.056	0.035
**CLX**	1.4	34.8	50.0	0.021
**VLX**	0.77	31.5	54.0	0.043
**SLT**	0.67	n.d.	0.374	0.009
**SAC**	6.2	n.d.	18.54	5.959
**HCT**	8.5	n.d.	0.328	0.29
**FAM**	>10	n.d.	0.922 ^c^	0.058 ^c^
**DCP**	3.06	0.346	1.2	0.038
**AAZ**	0.1	76.0	0.25	0.012

* Mean from three different assays, by a stopped flow technique (errors were in the range of ± 5–10% of the reported values).^a^ From ref. 24; ^b^ From ref. 39; ^c^ From ref. 40; n.d.: not detected.

## References

[1] Grimalt R. (2007). A practical guide to scalp disorders. J. Investig. Dermatol. Symp. Proc..

[2] Borda L.J., Wikramanayake T.C. (2015). Seborrheic Dermatitis and Dandruff: A Comprehensive Review. J. Clin. Investig. Dermatol..

[3] Karakadze M.A., Hirt P.A., Wikramanayake T.C. (2018). The genetic basis of seborrhoeic dermatitis: A review. J. Eur. Acad. Dermatol. Venereol..

[4] Paulino L.C. (2017). New perspectives on dandruff and seborrheic dermatitis: Lessons we learned from bacterial and fungal skin microbiota. Eur. J. Dermatol..

[5] Clavaud C., Jourdain R., Bar-Hen A., Tichit M., Bouchier C., Pouradier F., El Rawadi C., Guillot J., Menard-Szczebara F., Breton L. (2013). Dandruff is associated with disequilibrium in the proportion of the major bacterial and fungal populations colonizing the scalp. PLoS ONE.

[6] Turner G.A., Hoptroff M., Harding C.R. (2012). Stratum corneum dysfunction in dandruff. Int. J. Cosmet. Sci..

[7] Hay R.J. (2011). Malassezia, dandruff and seborrhoeic dermatitis: An overview. Br. J. Dermatol..

[8] Del Prete S., Vullo D., Ghobril C., Hitce J., Clavaud C., Marat X., Capasso C., Supuran C.T. (2019). Cloning, Purification, and Characterization of a beta-Carbonic Anhydrase from *Malassezia restricta*, an Opportunistic Pathogen Involved in Dandruff and Seborrheic Dermatitis. Int. J. Mol. Sci..

[9] Del Prete S., De Luca V., Vullo D., Osman S.M., AlOthman Z., Carginale V., Supuran C.T., Capasso C. (2016). A new procedure for the cloning, expression and purification of the beta-carbonic anhydrase from the pathogenic yeast *Malassezia globosa*, an anti-dandruff drug target. J. Enzym. Inhib. Med. Chem..

[10] Alterio V., Di Fiore A., D’Ambrosio K., Supuran C.T., De Simone G. (2012). Multiple binding modes of inhibitors to carbonic anhydrases: How to design specific drugs targeting 15 different isoforms?. Chem. Rev..

[11] Supuran C.T., Capasso C. (2017). An Overview of the Bacterial Carbonic Anhydrases. Metabolites.

[12] Ozensoy Guler O., Capasso C., Supuran C.T. (2016). A magnificent enzyme superfamily: Carbonic anhydrases, their purification and characterization. J. Enzym. Inhib. Med. Chem..

[13] Jensen E.L., Clement R., Kosta A., Maberly S.C., Gontero B. (2019). A new widespread subclass of carbonic anhydrase in marine phytoplankton. ISME J..

[14] Kikutani S., Nakajima K., Nagasato C., Tsuji Y., Miyatake A., Matsuda Y. (2016). Thylakoid luminal theta-carbonic anhydrase critical for growth and photosynthesis in the marine diatom *Phaeodactylum tricornutum*. Proc. Natl. Acad. Sci. USA.

[15] Capasso C., Supuran C.T. (2016). An Overview of the Carbonic Anhydrases from Two Pathogens of the Oral Cavity: *Streptococcus mutans* and *Porphyromonas gingivalis*. Curr. Top. Med. Chem..

[16] Capasso C., Supuran C.T. (2015). An overview of the alpha-, beta- and gamma-carbonic anhydrases from Bacteria: Can bacterial carbonic anhydrases shed new light on evolution of bacteria?. J. Enzym. Inhib. Med. Chem..

[17] Capasso C., Supuran C.T. (2015). An Overview of the Selectivity and Efficiency of the Bacterial Carbonic Anhydrase Inhibitors. Curr. Med. Chem..

[18] Prete S.D., Angeli A., Ghobril C., Hitce J., Clavaud C., Marat X., Supuran C.T., Capasso C. (2019). Anion Inhibition Profile of the beta-Carbonic Anhydrase from the Opportunist Pathogenic Fungus *Malassezia Restricta* Involved in Dandruff and Seborrheic Dermatitis. Metabolites.

[19] Nocentini A., Bua S., Del Prete S., Heravi Y.E., Saboury A.A., Karioti A., Bilia A.R., Capasso C., Gratteri P., Supuran C.T. (2018). Natural Polyphenols Selectively Inhibit beta-Carbonic Anhydrase from the Dandruff-Producing Fungus *Malassezia globosa*: Activity and Modeling Studies. Chem. Med. Chem..

[20] Nocentini A., Vullo D., Del Prete S., Osman S.M., Alasmary F.A.S., AlOthman Z., Capasso C., Carta F., Gratteri P., Supuran C.T. (2017). Inhibition of the beta-carbonic anhydrase from the dandruff-producing fungus *Malassezia globosa* with monothiocarbamates. J. Enzym. Inhib. Med. Chem..

[21] Entezari Heravi Y., Bua S., Nocentini A., Del Prete S., Saboury A.A., Sereshti H., Capasso C., Gratteri P., Supuran C.T. (2017). Inhibition of *Malassezia globosa* carbonic anhydrase with phenols. Bioorg. Med. Chem..

[22] Capasso C., Supuran C.T. (2015). Bacterial, fungal and protozoan carbonic anhydrases as drug targets. Expert Opin. Ther. Targets.

[23] Klengel T., Liang W.J., Chaloupka J., Ruoff C., Schroppel K., Naglik J.R., Eckert S.E., Mogensen E.G., Haynes K., Tuite M.F. (2005). Fungal adenylyl cyclase integrates CO_2_ sensing with cAMP signaling and virulence. Curr. Biol..

[24] Hewitson K.S., Vullo D., Scozzafava A., Mastrolorenzo A., Supuran C.T. (2012). Molecular cloning, characterization, and inhibition studies of a beta-carbonic anhydrase from *Malassezia globosa*, a potential antidandruff target. J. Med. Chem..

[25] Stalhberger T., Simenel C., Clavaud C., Eijsink V.G., Jourdain R., Delepierre M., Latge J.P., Breton L., Fontaine T. (2014). Chemical organization of the cell wall polysaccharide core of *Malassezia restricta*. J. Biol. Chem..

[26] Morand S.C., Bertignac M., Iltis A., Kolder I., Pirovano W., Jourdain R., Clavaud C. (2019). Complete Genome Sequence of *Malassezia restricta* CBS 7877, an Opportunist Pathogen Involved in Dandruff and Seborrheic Dermatitis. Microbiol. Resour. Announc..

[27] Angeli A., Pinteala M., Maier S.S., Del Prete S., Capasso C., Simionescu B.C., Supuran C.T. (2019). Inhibition of alpha-, beta-, gamma-, delta-, zeta- and eta-class carbonic anhydrases from bacteria, fungi, algae, diatoms and protozoans with famotidine. J. Enzym. Inhib. Med. Chem..

[28] Elleuche S., Poggeler S. (2010). Carbonic anhydrases in fungi. Microbiology.

[29] Vullo D., Lehneck R., Poggeler S., Supuran C.T. (2018). Sulfonamide inhibition studies of two beta-carbonic anhydrases from the ascomycete fungus *Sordaria macrospora*, CAS1 and CAS2. J. Enzym. Inhib. Med. Chem..

[30] Del Prete S., Vullo D., Osman S.M., AlOthman Z., Capasso C., Supuran C.T. (2015). Anion inhibition studies of the dandruff-producing fungus *Malassezia globosa* beta-carbonic anhydrase MgCA. Bioorg. Med. Chem. Lett..

[31] Singh S., Supuran C.T. (2016). In silico modeling of beta-carbonic anhydrase inhibitors from the fungus *Malassezia globosa* as antidandruff agents. J. Enzym. Inhib. Med. Chem..

[32] Schlicker C., Hall R.A., Vullo D., Middelhaufe S., Gertz M., Supuran C.T., Muhlschlegel F.A., Steegborn C. (2009). Structure and inhibition of the CO_2_-sensing carbonic anhydrase Can2 from the pathogenic fungus *Cryptococcus neoformans*. J. Mol. Biol..

[33] Supuran C.T. (2016). How many carbonic anhydrase inhibition mechanisms exist?. J. Enzym. Inhib. Med. Chem..

[34] Monti S.M., Maresca A., Viparelli F., Carta F., De Simone G., Muhlschlegel F.A., Scozzafava A., Supuran C.T. (2012). Dithiocarbamates are strong inhibitors of the beta-class fungal carbonic anhydrases from *Cryptococcus neoformans*, *Candida albicans* and *Candida glabrata*. Bioorg. Med. Chem. Lett..

[35] Carta F., Innocenti A., Hall R.A., Muhlschlegel F.A., Scozzafava A., Supuran C.T. (2011). Carbonic anhydrase inhibitors. Inhibition of the beta-class enzymes from the fungal pathogens *Candida albicans* and *Cryptococcus neoformans* with branched aliphatic/aromatic carboxylates and their derivatives. Bioorg. Med. Chem. Lett..

[36] Vullo D., Del Prete S., Nocentini A., Osman S.M., AlOthman Z., Capasso C., Bozdag M., Carta F., Gratteri P., Supuran C.T. (2017). Dithiocarbamates effectively inhibit the beta-carbonic anhydrase from the dandruff-producing fungus *Malassezia globosa*. Bioorg. Med. Chem..

[37] De Luca V., Del Prete S., Supuran C.T., Capasso C. (2015). Protonography, a new technique for the analysis of carbonic anhydrase activity. J. Enzym. Inhib. Med. Chem..

[38] Guzel O., Maresca A., Hall R.A., Scozzafava A., Mastrolorenzo A., Muhlschlegel F.A., Supuran C.T. (2010). Carbonic anhydrase inhibitors. The beta-carbonic anhydrases from the fungal pathogens *Cryptococcus neoformans* and *Candida albicans* are strongly inhibited by substituted-phenyl-1*H*-indole-5-sulfonamides. Bioorg. Med. Chem. Lett..

[39] Supuran C.T. (2008). Carbonic anhydrases: Novel therapeutic applications for inhibitors and activators. Nat. Rev. Drug. Discov..

[40] Angeli A., Ferraroni M., Supuran C.T. (2018). Famotidine, an Antiulcer Agent, Strongly Inhibits *Helicobacter pylori* and Human Carbonic Anhydrases. ACS Med. Chem. Lett..

[41] Supuran C.T. (2017). Advances in structure-based drug discovery of carbonic anhydrase inhibitors. Expert Opin. Drug. Discov..

[42] Vullo D., Del Prete S., Fisher G.M., Andrews K.T., Poulsen S.A., Capasso C., Supuran C.T. (2015). Sulfonamide inhibition studies of the eta-class carbonic anhydrase from the malaria pathogen *Plasmodium falciparum*. Bioorg. Med. Chem..

[43] Vullo D., De Luca V., Del Prete S., Carginale V., Scozzafava A., Capasso C., Supuran C.T. (2015). Sulfonamide inhibition studies of the gamma-carbonic anhydrase from the Antarctic bacterium *Pseudoalteromonas haloplanktis*. Bioorg. Med. Chem. Lett..

[44] Vullo D., De Luca V., Del Prete S., Carginale V., Scozzafava A., Capasso C., Supuran C.T. (2015). Sulfonamide inhibition studies of the gamma-carbonic anhydrase from the Antarctic cyanobacterium *Nostoc commune*. Bioorg. Med. Chem..

[45] Dedeoglu N., DeLuca V., Isik S., Yildirim H., Kockar F., Capasso C., Supuran C.T. (2015). Sulfonamide inhibition study of the beta-class carbonic anhydrase from the caries producing pathogen *Streptococcus mutans*. Bioorg. Med. Chem. Lett..

[46] Alafeefy A.M., Ceruso M., Al-Tamimi A.M., Del Prete S., Supuran C.T., Capasso C. (2015). Inhibition studies of quinazoline-sulfonamide derivatives against the gamma-CA (PgiCA) from the pathogenic bacterium, *Porphyromonas gingivalis*. J. Enzym. Inhib. Med. Chem..

[47] Alafeefy A.M., Abdel-Aziz H.A., Vullo D., Al-Tamimi A.M., Awaad A.S., Mohamed M.A., Capasso C., Supuran C.T. (2015). Inhibition of human carbonic anhydrase isozymes I, II, IX and XII with a new series of sulfonamides incorporating aroylhydrazone-, [1,2,4] triazolo [3,4-b] [1,3,4]t hiadiazinyl- or 2-(cyanophenylmethylene)-1, 3, 4-thiadiazol-3(2H)-yl moieties. J. Enzym. Inhib. Med. Chem..

[48] Diaz J.R., Fernandez Baldo M., Echeverria G., Baldoni H., Vullo D., Soria D.B., Supuran C.T., Cami G.E. (2016). A substituted sulfonamide and its Co (II), Cu (II), and Zn (II) complexes as potential antifungal agents. J. Enzym. Inhib. Med. Chem..

[49] Del Prete S., Vullo D., De Luca V., Carginale V., Osman S.M., AlOthman Z., Supuran C.T., Capasso C. (2016). Comparison of the sulfonamide inhibition profiles of the alpha-, beta- and gamma-carbonic anhydrases from the pathogenic bacterium *Vibrio cholerae*. Bioorg. Med. Chem. Lett..

[50] Del Prete S., Vullo D., De Luca V., Carginale V., Osman S.M., AlOthman Z., Supuran C.T., Capasso C. (2016). Cloning, expression, purification and sulfonamide inhibition profile of the complete domain of the eta-carbonic anhydrase from *Plasmodium falciparum*. Bioorg. Med. Chem. Lett..

[51] Del Prete S., Vullo D., De Luca V., Carginale V., Ferraroni M., Osman S.M., AlOthman Z., Supuran C.T., Capasso C. (2016). Sulfonamide inhibition studies of the beta-carbonic anhydrase from the pathogenic bacterium *Vibrio cholerae*. Bioorg. Med. Chem..

[52] Abdel Gawad N.M., Amin N.H., Elsaadi M.T., Mohamed F.M., Angeli A., De Luca V., Capasso C., Supuran C.T. (2016). Synthesis of 4-(thiazol-2-ylamino)-benzenesulfonamides with carbonic anhydrase I, II and IX inhibitory activity and cytotoxic effects against breast cancer cell lines. Bioorg. Med. Chem..

[53] Supuran C.T. (2016). Legionella pneumophila Carbonic Anhydrases: Underexplored Antibacterial Drug Targets. Pathogens.

[54] Nishimori I., Vullo D., Minakuchi T., Scozzafava A., Capasso C., Supuran C.T. (2014). Sulfonamide inhibition studies of two beta-carbonic anhydrases from the bacterial pathogen *Legionella pneumophila*. Bioorg. Med. Chem..

[55] Vullo D., Sai Kumar R.S., Scozzafava A., Capasso C., Ferry J.G., Supuran C.T. (2013). Anion inhibition studies of a beta-carbonic anhydrase from Clostridium perfringens. Bioorg. Med. Chem. Lett..

[56] Nishimori I., Minakuchi T., Maresca A., Carta F., Scozzafava A., Supuran C.T. (2010). The beta-carbonic anhydrases from *Mycobacterium tuberculosis* as drug targets. Curr. Pharm. Des..

[57] Carta F., Maresca A., Covarrubias A.S., Mowbray S.L., Jones T.A., Supuran C.T. (2009). Carbonic anhydrase inhibitors. Characterization and inhibition studies of the most active beta-carbonic anhydrase from *Mycobacterium tuberculosis*, Rv3588c. Bioorg. Med. Chem. Lett..

[58] Supuran C.T. (2016). Structure and function of carbonic anhydrases. Biochem. J..

[59] Supuran C.T. (2016). Carbonic anhydrase inhibition and the management of neuropathic pain. Expert Rev. Neurother..

[60] Supuran C.T. (2016). Drug interaction considerations in the therapeutic use of carbonic anhydrase inhibitors. Expert Opin. Drug. Metab. Toxicol..

[61] Bradford M.M. (1976). A rapid and sensitive method for the quantitation of microgram quantities of protein utilizing the principle of protein-dye binding. Anal. Biochem..

[62] Laemmli U.K. (1970). Cleavage of structural proteins during the assembly of the head of bacteriophage T4. Nature.

[63] Del Prete S., De Luca V., Iandolo E., Supuran C.T., Capasso C. (2015). Protonography, a powerful tool for analyzing the activity and the oligomeric state of the gamma-carbonic anhydrase identified in the genome of *Porphyromonas gingivalis*. Bioorg. Med. Chem..

[64] Del Prete S., De Luca V., Supuran C.T., Capasso C. (2015). Protonography, a technique applicable for the analysis of eta-carbonic anhydrase activity. J. Enzym. Inhib. Med. Chem..

[65] Del Prete S., Vullo D., Caminiti-Segonds N., Zoccola D., Tambutte S., Supuran C.T., Capasso C. (2018). Protonography and anion inhibition profile of the alpha-carbonic anhydrase (CruCA4) identified in the Mediterranean red coral *Corallium rubrum*. Bioorg. Chem..

[66] Khalifah R.G. (1971). The carbon dioxide hydration activity of carbonic anhydrase. I. Stop-flow kinetic studies on the native human isoenzymes B and C. J. Biol. Chem..

[67] Del Prete S., Vullo D., De Luca V., Carginale V., di Fonzo P., Osman S.M., AlOthman Z., Supuran C.T., Capasso C. (2016). Anion inhibition profiles of alpha-, beta- and gamma-carbonic anhydrases from the pathogenic bacterium *Vibrio cholerae*. Bioorg. Med. Chem..

[68] Del Prete S., Vullo D., De Luca V., Carginale V., di Fonzo P., Osman S.M., AlOthman Z., Supuran C.T., Capasso C. (2016). Anion inhibition profiles of the complete domain of the eta-carbonic anhydrase from *Plasmodium falciparum*. Bioorg. Med. Chem..

[69] De Luca V., Vullo D., Del Prete S., Carginale V., Osman S.M., AlOthman Z., Supuran C.T., Capasso C. (2016). Cloning, characterization and anion inhibition studies of a gamma-carbonic anhydrase from the Antarctic bacterium *Colwellia psychrerythraea*. Bioorg. Med. Chem..

